# Service-User Led Recommendations on Improving Medium to Low Secure Unit Transfer Experiences in East London During COVID-19; a Quality Improvement Project

**DOI:** 10.1192/bjo.2022.309

**Published:** 2022-06-20

**Authors:** Hardeep Kandola, Erin Vignali, Xuezi Bai

**Affiliations:** 1 Barts and the London School of Medicine and Dentistry, London, United Kingdom; 2 East London NHS Foundation Trust, London, United Kingdom

## Abstract

**Aims:**

Forensic service-users (SU) are often transferred between units at short notice, with little information about their new ward and unaddressed concerns. The findings of our first Plan, Do, Study, Act (PDSA) quality improvement project exploring SU's transfer experiences from medium to low secure units during COVID-19 in East London. Whilst unsettling, it may also impact therapeutic progress due to difficulties in developing new relationships with clinicians and ward residents. Our second PDSA cycle explores SU concerns and identifies improvement recommendations.

**Methods:**

Five SUs consented to take part in the experience-based co-design approach. Remote semi-structured interviews explored experiences and suggestions for improvement. Audio transcriptions were thematically analysed to assess information provision, transfer expectations, new ward perceptions, available support and overall reflections.

**Results:**

Recipients of a tour prior to moving felt most informed about the transfer and reported not requiring further information. Without the tour, some SUs were unaware of the name or type of their future ward. Therefore, SUs recommended everyone should have a tour prior to transfer, or where not possible a video tour. Two weeks was felt to be the ideal amount of notice prior to transfer and in the absence of this, weekly updates during ward round. Being able to talk to an SU who had recently moved to the new unit would be ideal, or in lieu of this a pre-recorded question and answer (Q&A) video.

SUs were interested in the new ward policy regarding leave and mobile phone criteria. A recurring issue involved SUs being promised unescorted leave on the new ward, but this not taking place. Importance was also placed on the family being updated during the transfer and moving with a preferred staff member. Many SUs felt the process was smooth yet held concerns about forming new friendships. They felt their psychologist, primary nurse or ward manager were easiest to approach with concerns. On the transfer day, SUs benefitted from the encouragement and congratulations from the old team, however cited feeling rushed when moving.

**Conclusion:**

Implementing a pre-recorded tour with unit facility highlights and a recent SU transfer Q&A video would improve the transfer experience. Having clear policies regarding leave and mobile phone criteria prior to moving improves clarity. Our approach to include SUs in the transfer meeting with both the new and old wards ensures transparent expectations. We continue to monitor the success of these interventions on transfer experience through further PDSA cycles.

